# Hemagglutinin Sequence Conservation Guided Stem Immunogen Design from Influenza A H3 Subtype

**DOI:** 10.3389/fimmu.2015.00329

**Published:** 2015-06-26

**Authors:** V. Vamsee Aditya Mallajosyula, Michael Citron, Francesca Ferrara, Nigel J. Temperton, Xiaoping Liang, Jessica A. Flynn, Raghavan Varadarajan

**Affiliations:** ^1^Molecular Biophysics Unit, Indian Institute of Science, Bangalore, India; ^2^Merck Research Laboratories, West Point, PA, USA; ^3^Viral Pseudotype Unit, Medway School of Pharmacy, University of Kent, Chatham, Kent, UK

**Keywords:** protein minimization, hemagglutinin stalk, subunit vaccine, pre-fusion conformation, antibody response, *Escherichia coli*

## Abstract

Seasonal epidemics caused by influenza A (H1 and H3 subtypes) and B viruses are a major global health threat. The traditional, trivalent influenza vaccines have limited efficacy because of rapid antigenic evolution of the circulating viruses. This antigenic variability mediates viral escape from the host immune responses, necessitating annual vaccine updates. Influenza vaccines elicit a protective antibody response, primarily targeting the viral surface glycoprotein hemagglutinin (HA). However, the predominant humoral response is against the hypervariable head domain of HA, thereby restricting the breadth of protection. In contrast, the conserved, subdominant stem domain of HA is a potential “universal” vaccine candidate. We designed an HA stem-fragment immunogen from the 1968 pandemic H3N2 strain (A/Hong Kong/1/68) guided by a comprehensive H3 HA sequence conservation analysis. The biophysical properties of the designed immunogen were further improved by C-terminal fusion of a trimerization motif, “isoleucine-zipper”, or “foldon”. These immunogens elicited cross-reactive, antiviral antibodies and conferred partial protection against a lethal, homologous HK68 virus challenge *in vivo*. Furthermore, bacterial expression of these immunogens is economical and facilitates rapid scale-up.

## Introduction

Influenza (flu) virus infection causes respiratory illness in humans. Preventive vaccination is the best way of controlling influenza infections (1). Antiviral medications such as oseltamivir, zanamivir, and peramivir are used to treat influenza infections (2, 3). Additionally, the application of human monoclonal antibodies in therapeutic treatment of influenza infections is also being explored (4–7).

The rapidly evolving influenza viruses are diverse and have been categorized into three immunological types: A, B, and C. The influenza A viruses are further classified on the basis of their surface glycoproteins, hemagglutinin (HA) and neuraminidase (NA), into 18 HA and 11 NA subtypes (8). H17 and H18 HAs are putative HA-like molecules, since their hemagglutination activity has not been established. NA activity of N10 and N11 NAs has also not been shown. Antigenic relatedness within HA facilitates clustering influenza A viruses into two major phylogenetic groups: group 1 (subtypes: H1, H2, H5, H6, H8, H9, H11, H12, H13, H16, H17, and H18) and group 2 (subtypes: H3, H4, H7, H10, H14, and H15) (8). Currently, only influenza A (H1 and H3 subtypes) and B viruses cause seasonal epidemics in humans (9). However, the perceived threat of highly pathogenic avian influenza viruses (H5N1) and new reports of influenza strains (H7N9, H6N1, and H10N8) crossing over the species barrier and infecting humans (10–13) necessitate the development of a “universal” influenza vaccine.

Currently, there are two variants of influenza vaccine: inactivated influenza vaccine (IIV) and live attenuated influenza vaccine (LAIV). The efficacy of IIV and LAIV in children and adults has been extensively evaluated (14, 15). All commercially available influenza vaccines are manufactured by propagation of virus in embryonated chicken eggs with a production time of 6–8 months, except the trivalent recombinant influenza vaccine [RIV3] (FluBlok, Protein Sciences) and cell culture-based IIV [ccIIV3] (Flucelvax, Novartis)^1^. Therefore, manufacturing large amounts of vaccine at short notice during an epidemic/pandemic is difficult. Furthermore, preparedness against influenza infection is compromised due to the lack of a foolproof method for the annual selection of vaccine strains (16).

Hemagglutinin is highly immunogenic and its efficacy as a stand-alone vaccine candidate has been extensively investigated (1, 17). The precursor polypeptide (HA0) oligomerizes in the endoplasmic reticulum (ER) and is transported to the cell surface via the Golgi apparatus (18). HA0 is subsequently cleaved by cellular proteases into the disulfide-linked, fusion competent HA1 and HA2 subunits (19). The immunodominant, membrane distal, globular head domain of HA containing the receptor-binding site (RBS) is assembled exclusively by the HA1 subunit, while the viral membrane proximal, stem domain of HA is comprised primarily of the HA2 subunit (20).

A comprehensive analysis of H3 HA sequences revealed a high degree of conservation within the stem domain as opposed to the variable head domain, in agreement with published results (21, 22). The degree of conservation is inversely correlated with antigenic distance between influenza strains. As shown previously, the degree of overall residue conservation in HA within a subtype is significantly greater than group-specific residue conservation (1, 21), and therefore eliciting pan-influenza neutralizing antibodies has remained elusive. The dominant antibody response post-vaccination is against the variable head domain of HA, thereby limiting vaccine efficacy. However, isolation of several broadly neutralizing antibodies (bnAbs) targeting conserved epitopes in the HA stem (4, 5, 23–27) has facilitated efforts in developing stem-based vaccine candidates with the potential to confer hetero-subtypic protection (1). Nonetheless, it has been extremely challenging to activate stem-directed bnAbs in humans because of their low frequency in the influenza-specific memory B-cell pool (21). The metastable conformation of the HA2 subunit in the pre-fusion state of HA further compounds the difficulty in exploiting the conserved epitopes of the HA stem in developing a “universal” vaccine. Diverse strategies have been adopted to express the HA stem in the pre-fusion conformation (28–32). We have previously demonstrated the soluble expression of HA stem-fragments in *Escherichia coli* (*E. coli*) by maintaining the interaction network within the HA stem and introducing designed mutations. These immunogens conferred robust subtype-specific and modest hetero-subtypic protection *in vivo* against influenza A group 1 viruses (32).

Structural analysis of the HA stem reveals differences at the N-terminus of the long alpha helix (LAH) and the composition of ionizable residues proximal to the fusion peptide between influenza A phylogenetic groups 1 and 2 (33). In order to mitigate the threat of circulating influenza A viruses from these distinct structural classes (H1 from group 1 and H3 from group 2), we characterized an HA stem-fragment immunogen (H3HA10) from the H3N2 strain (A/Hong Kong/1/68), which caused the “1968 influenza pandemic.” We evaluated the effect of trimerization motifs, the coiled-coil isoleucine zipper (IZ) (34) and the globular, β-rich “foldon” (35), belonging to disparate structural classes as a C-terminal fusion to H3HA10. The oligomeric derivatives of H3HA10 had improved biophysical properties and elicited cross-reactive, antiviral antibodies in mice. The elicited antibodies inhibited the entry of a heterologous H3 HA pseudotyped virus *in vitro*. These immunogens conferred partial protection against a lethal, homologous HK68 virus challenge *in vivo*. Additionally, bacterial expression of these immunogens is cost-effective and enables rapid production.

## Materials and Methods

### Sequence analysis

All non-identical, full-length H3 HA protein sequences (3169 sequences) reported from human hosts were retrieved from the NCBI-Flu Database^2^. These H3 HA sequences were subsequently clustered at 99% homology using Cluster Database at High Identity with Tolerance (CD-HIT) (36) to filter-out 392 unique, representative sequences, which were then multiply aligned using CLUSTAL (37). The alignment file lists a quality score for every position in the protein sequence, which is a measure of residue conservation. The quality scores were then binned and mapped onto the crystal structure of H3N2 A/Hong Kong/1/68 HA [protein data bank (PDB) ID: 1HGD (38)].

### Protein expression and purification

The *E. coli* codon-optimized gene sequence of our designed construct H3HA10 was synthesized with a stop codon at the 3’ end (GenScript, USA). The gene was cloned into the expression vector pET-28a (+) (Novagen) in-frame with the N-terminal His-tag between the *Nde*I and *Bam*HI restriction sites. The *E. coli* codon-optimized gene sequences encoding IZ and foldon were individually synthesized (Abexome, India) with *Kpn*I and *Hin*dIII restriction sites at the 5’ and 3’ ends respectively. In order to facilitate the cloning of a trimerization motif at the C-terminus of H3HA10 to generate H3HA10-IZ and H3HA10-Foldon, the stop codon in H3HA10 at the 3’ end was mutated and a unique *Kpn*I restriction site was generated using site-directed mutagenesis. All the generated clones were confirmed by sequencing (Macrogen, South Korea).

The designed proteins were expressed in *E. coli* BL21(DE3) cells. H3HA10, H3HA10-IZ, and H3HA10-Foldon were all purified using a similar protocol from the soluble fraction of the cell culture lysate. Briefly, a single transformed colony of *E. coli* BL21(DE3) cells was inoculated into 50 ml of Luria-Bertani broth (HiMedia). The primary culture (50 ml) was grown overnight until saturation at 37°C. Subsequently, 2 L of Luria-Bertani broth (500 ml × 4) was inoculated with 1% of the saturated primary inoculum and grown at 37°C until an OD_600_ of ~0.6–0.8 was reached. The cultures were then induced with 1 mM isopropyl-β-thiogalactopyranoside (IPTG). The cells were grown for another 12–16 h at 20°C post-induction. Next, the culture was spun down at 5000 × *g* for 15 min at 4°C. The pelleted cells were resuspended in 100 ml of phosphate-buffered saline (PBS, pH 7.4). The cell suspension was lysed by sonication and subsequently centrifuged at 14,000 × *g* for 45 min at 4°C. The supernatant from the cell culture lysate was incubated with buffer-equilibrated Ni-NTA resin (GE HealthCare) for 2–3 h at 4°C to facilitate binding. The protein was eluted in 2 ml fractions using an imidazole gradient (in PBS, pH 7.4). The eluted fractions were analyzed by SDS-PAGE and pooled for dialysis against PBS (pH 7.4) containing 1 mM EDTA. The dialyzed protein was concentrated to a final concentration of ~5 mg/ml and its identity was confirmed by electrospray ionization-mass spectroscopy (ESI-MS).

### Fluorescence spectroscopy

The intrinsic fluorescence measurements for all proteins were recorded at 25°C on a Jasco FP-6300 spectrofluorometer. The protein samples (concentration of 1–3 μM) were excited at a wavelength of 280 nm, and emission was monitored between 300 and 400 nm. The spectrofluorometer slit widths for excitation and emission were set at 3 and 5 nm, respectively. The represented fluorescence signals were averaged over five consecutive scans and corrected for buffer signals. The fluorescence signal of the native protein was recorded in PBS (pH 7.4). The protein was denatured in 7M guanidine hydrochloride (GdmCl) to record the fluorescence signal in the unfolded state.

### NMR spectroscopy

One-dimensional ^1^H NMR spectra of all the protein samples were recorded at 25°C on an Agilent 600 MHz NMR spectrometer. The spectra were recorded using a triple resonance cryo-probe fitted with a *z*-axis only pulsed field gradient accessory. External DSS was used for referencing ^1^H chemical shifts. A spectral width of 9615.4 Hz was sampled. The excitation sculpting pulse scheme was used to achieve solvent suppression (39). All the protein samples were prepared in PBS (pH 7.4) (90% H_2_O:10% D_2_O). A total of 1024 scans were recorded with a 1 s relaxation delay.

### Gel filtration chromatography

The purified proteins were analyzed by gel filtration chromatography on a Superdex-200 analytical gel filtration column (GE HealthCare) equilibrated with PBS (pH 7.4) to determine their oligomeric state under native conditions. The column was calibrated using a broad range of molecular weight markers as indicated (GE HealthCare).

### Antibody binding studies using surface plasmon resonance

Binding affinity of the purified proteins (H3HA10, H3HA10-IZ, and H3HA10-Foldon) and full-length recombinant HAs (rHAs) (H3N2 A/Aichi/2/68 and H1N1 A/Puerto Rico/8/34) (Sino Biological Inc., Beijing, China) to the single-chain variable fragment (scFv) of the stem-directed bnAb FI6v3 was determined by surface plasmon resonance (SPR) experiments performed with a Biacore2000 optical biosensor (Biacore, Uppsala, Sweden) at 25°C. Recombinant FI6v3-scFv was produced as described previously (32). Amine coupling chemistry was used to immobilize 750 response units (RU) of the ligand (FI6v3-scFv) to the surface of a CM5 chip (GE HealthCare, Uppsala, Sweden). A sensor channel immobilized with ovalbumin served as a negative-control for each binding interaction. A concentration series of each analyte were passed over the ligand(s) in a running buffer of PBS (pH 7.4) with 0.05% P20 surfactant at a flow rate of 30 μl/min to determine the binding kinetics. The sensor surface was regenerated with 2M MgCl_2_ after every binding event. The kinetic data was obtained using the concentration of the monomer for H3HA10 and the concentration(s) of the trimer(s) for H3HA10-IZ, H3HA10-Foldon, and rHAs. The concentration used for fitting the kinetic data was in accordance with the oligomeric state of the protein as determined by gel filtration chromatography. The kinetic parameters of binding were obtained by a global fitting of the data to the simple 1:1 Langmuir interaction model using BIA EVALUATION 3.1 software.

### Immunization and virus challenge studies

The female BALB/c mice (4–5 weeks old) (Taconic Farms, Inc., Germantown, NY, USA) used in this study were maintained at the animal facilities of Merck Research Laboratories. The animal study was approved by the Merck Research Laboratories Institutional Animal Care and Use Committee. Each group of mice (*n* = 10) were immunized intramuscularly with 20 μg of the test immunogen along with 100 μg CpG7909 adjuvant (TriLink BioTechnologies, San Diego, CA, USA) at days 0 (prime) and 28 (boost). Naïve mice were used as controls. Serum samples were obtained from all the mice 3 weeks after prime and 2 weeks after boost by tail venipuncture and collected in Microtainer serum separator tubes (BD Biosciences, Franklin Lakes, NJ, USA). About 3 weeks after the boost, mice were anesthetized with ketamine/xylazine and challenged intranasally with 2LD_90_ (lethal dose) of mouse-adapted H3N2 A/HK/1/68 virus in 20 μL of PBS. The mice were monitored for survival and weight change for 14 days post virus challenge.

### Determination of serum antibody titers

The serum antibody-titers against test immunogens (H3HA10, H3HA10-IZ, and H3HA10-Foldon) were determined by ELISA. Briefly, 200 ng of the protein was coated on 96-well Nunc plates (Thermo Fisher Scientific, Rochester, NY, USA) and incubated overnight at 4°C. Next, the plates were washed with PBS containing 0.05%Tween-20 (PBST) and blocked with 1% bovine serum albumin (BSA) (Sigma) in PBST (PBSB) for 1 h. Antiserum raised against the test immunogen was fourfold serially diluted in PBSB and added to each well. The plates were washed with PBST after 2 h of incubation with sera at room temperature. Fifty microliters of alkaline phosphatase (ALP)-conjugated goat anti-mouse secondary antibody (Sigma) used at a predetermined dilution of 1:10000 in PBSB were added to each well and incubated at room temperature for 2 h. After washing, the plates were developed using the chromogenic substrate p-nitrophenyl phosphate (Sigma) and read at 405 nm (SPECTRAmax Plus 384, Molecular Devices, USA). Antibody titer was determined as the reciprocal of the highest serum dilution that gave an OD_405_ value above the mean + 2 × SD of control wells.

### Binding of antisera to full-length recombinant HAs

The binding of antibodies elicited by the test immunogens to a panel of full-length rHA proteins was determined by ELISA. Briefly, 200 ng of mammalian-cell expressed rHA proteins (H3N2 A/Aichi/2/68, H3N2 A/Brisbane/10/07, H7N7 A/chicken/Netherlands/1/03, H1N1 A/Puerto Rico/8/34, and H1N1 A/California/04/2009 from Sino Biological Inc., Beijing, China) were coated on 96-well Nunc plates (Thermo Fisher Scientific, Rochester, NY, USA) and incubated overnight at 4°C. Ovalbumin (200 ng/well) coated wells were used as a control for non-specific binding. Plates were washed with PBST and subsequently blocked with PBSB. The rHA proteins were then incubated for 2 h with a fourfold serial dilution of the antisera (starting at a dilution of 1:100). The plates were then washed with PBST and incubated for 2 h at room temperature with ALP-conjugated goat anti-mouse secondary antibody (Sigma) used at a dilution of 1:10000. After another round of washing, the plates were developed with the chromogenic substrate p-nitrophenyl phosphate (Sigma). The antibody titer against rHA proteins was determined as the reciprocal of the highest serum dilution that gave an OD_405_ (SPECTRAmax Plus 384, Molecular Devices, USA) value above the mean + 2 × SD of control wells.

### Pseudotyped virus particle entry inhibition assay

The antisera raised against the designed immunogens were tested in a pseudotype virus particle entry inhibition assay as described previously (40, 41). Briefly, HIV gag-pol plasmid p8.91, firefly luciferase expressing plasmid pCSFLW, H3 HA (A/Wisconsin/67/2005) expressing plasmid, NA expressing plasmid (A/Udorn/307/1972 N2), and pCAGGS-HAT (42) expressing plasmid were co-transfected into human embryonic kidney (HEK 293T/17) cells using Fugene-6 transfection reagent (Promega) and incubated for 24 h. The supernatant containing the released pseudotypes was harvested 48 h post-infection, filtered through a 0.45 μm filter, and stored at −80°C until further use.

Serial dilutions of the antisera were incubated with 2 × 10^7^ relative luminescence units (RLUs) of pseudotypes/well in 96-well flat-bottomed white plates (Nunc) in a final volume of 50 μl at 37°C for 1 h. After the incubation, 1.5 × 10^4^ HEK293T cells were added to each well. The plates were subsequently incubated for another 48 h at 37°C. The luminescence signals were assayed using the Bright-Glo assay system (Promega). The half-maximal inhibitory concentrations (IC_50_) of entry inhibition were determined using Prism v6.0 (GraphPad Software).

### Statistical analysis

The differences in antibody/entry inhibition titers were analyzed by analysis of variance and Student’s *t*-test. The fractional body weight of mice was calculated relative to their initial body weight. Differences in the mean fractional body weight of surviving mice were analyzed using analysis of variance and Student’s *t*-test. Kaplan–Meier survival analysis with the log rank significance test was used to analyze the difference in survival across groups.

## Results

### Design of HA stem-fragment immunogens

**Figure 1:**
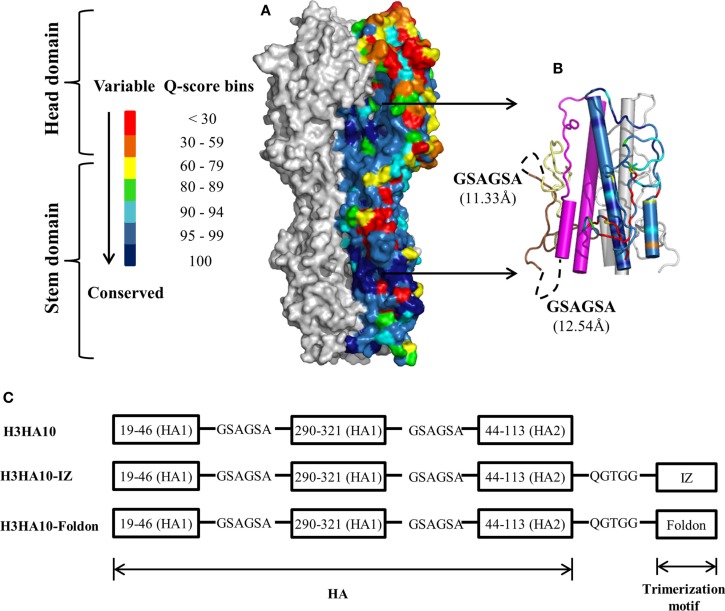
**HA sequence conservation guided immunogen design**. **(A)** The residue conservation among H3 HA human isolates is mapped onto a surface representation of H3N2 A/Hong Kong/1/68 HA trimer [PDB ID: 1HGD (38)]. The quality score (Q-score) at every position in the protein sequence, which is a measure of residue conservation, was obtained from a multiple sequence alignment of H3 HA sequences (*n* = 3169) and binned. The HA stem is well conserved within a subtype. One monomer is colored according to the Q-score scale. Rest of HA (gray). **(B)** Conserved HA stem-fragments are “stitched” together in H3HA10 (cartoon). One monomer is colored according to the Q-score scale [indicated in **(A)**] to highlight the residue conservation in the designed immunogen. Stable structural breakpoints with optimal Cα–Cα distances (shown in another monomer) were determined by analysis of the H3 HA distance matrix. Soluble, flexible linker as indicated (black, dashed curve) was used to connect the HA1-subunit fragment 19_1_-46_1_ (pale yellow) to 290_1_-321_1_ (brown). A 6-residue linker (GSAGSA) connects the HA1 (brown) and HA2 (magenta) subunits in H3HA10. The third monomer is in gray. **(C)** Derivative(s) of H3HA10 were made with C-terminal trimerization motif(s), IZ, and foldon (34, 35). The figures **(A,B)** were rendered using PyMOL.

Current influenza vaccines elicit a robust strain-specific antibody response which neutralizes the virus and confers protection (1). The primary response is against the immunodominant antigenic sites in the head domain of HA (43). However, the virus has evolved a mechanism of “antigenic drift” wherein mutations accumulated in these antigenic sites facilitate escape from the host immune pressure. Immune selection pressures coerce influenza virus into presenting a continually “moving target”. Therefore, we analyzed a large dataset of full-length H3 HA sequences (3169 sequences) reported from human hosts to identify conserved regions on HA. In agreement with previous results (1, 21, 22), the HA stem is on average more conserved than the variable head domain within a subtype (Figure 1A). However, there are pockets of high conservation even within the head domain of HA and monoclonal antibodies binding within the RBS have been isolated that neutralize drift variants of a HA subtype (6). The conservation within the HA stem has been ascribed to the less-than-optimal accessibility on the virion surface and the functional restraint imposed by conformational changes in the stem domain that are essential for infection. However, recent cryoEM studies of H1N1 A/California/7/2009 virus pre-incubated with the stem-directed bnAb C179 demonstrated that ~75% of HA trimers on the virion surface were in complex with the antibody (44). The study demonstrated that antibody binding to the HA stem on the virion is not severely impeded by accessibility.

A large fraction (≥90%) of the epitopes identified by the human B-cell population is conformation sensitive (45). In fact, extensive conformational rearrangement of the HA stem at low pH disrupts the epitope of HA stem-directed bnAbs. We analyzed the interaction network within H3 HA [H3N2 A/Hong Kong/1/68, PDB ID: 1HGD (38)] using PREDBURASA, as described previously (46), to identify HA stem fragments defined by stable structural breakpoints. During HA protein minimization, we performed iterative calculations to identify residue fragments: 19_1_-46_1_, 290_1_-321_1_, and 44_2_-113_2_ (included in H3HA10) within the stem domain having minimalistic interactions with the rest of HA (Figure 1B). Residues from HA1 and HA2 subunits are distinguished by subscripts 1 or 2 respectively. The termini of HA stem-fragments in H3HA10 also had optimal Cα-Cα distances for “molecular stitching” as indicated in Figure 1B from our analysis of the Cα-Cα distance matrix of H3 HA. These fragments were connected with flexible, soluble linkers as described previously (47). The loss of native contacts in H3HA10 as a consequence of protein minimization exposes previously buried hydrophobic patches which can potentially aggregate. We re-surfaced these hydrophobic patches with polar amino acid substitutions as described previously (32). We have previously designed stable influenza and HIV-1 immunogens using a similar approach (28, 30, 32, 48). The following mutations were introduced in H3HA10 to resurface the hydrophobic patches: V20_1_S, V297_1_T, I300_1_R, Y302_1_S, M320_1_Q, and I45_2_T. In the full-length H3 HA, Cys281_1_ and Cys305_1_ form an intramolecular disulfide bond. Since residue Cys281_1_ was not incorporated in H3HA10, we mutated Cys305_1_ to Ser to prevent the formation of incorrect, intermolecular disulfide bonds in the absence of its cognate partner (Cys281_1_). Aspartate mutations (F63_2_D and V73_2_D), previously shown to destabilize the low-pH conformation of HA (28) were also incorporated in H3HA10. We have previously shown that synthetic trimerization motifs promote the oligomerization of HA stem in the absence of the trans-membrane (TM) domain (32). We made derivatives of H3HA10 with the coiled-coil IZ (H3HA10-IZ) and the globular, β-rich “foldon” (H3HA10-Foldon) (Figure 1C). Figure S1 in Supplementary Material lists the sequences of all the designed constructs.

### Protein purification and characterization

**Figure 2:**
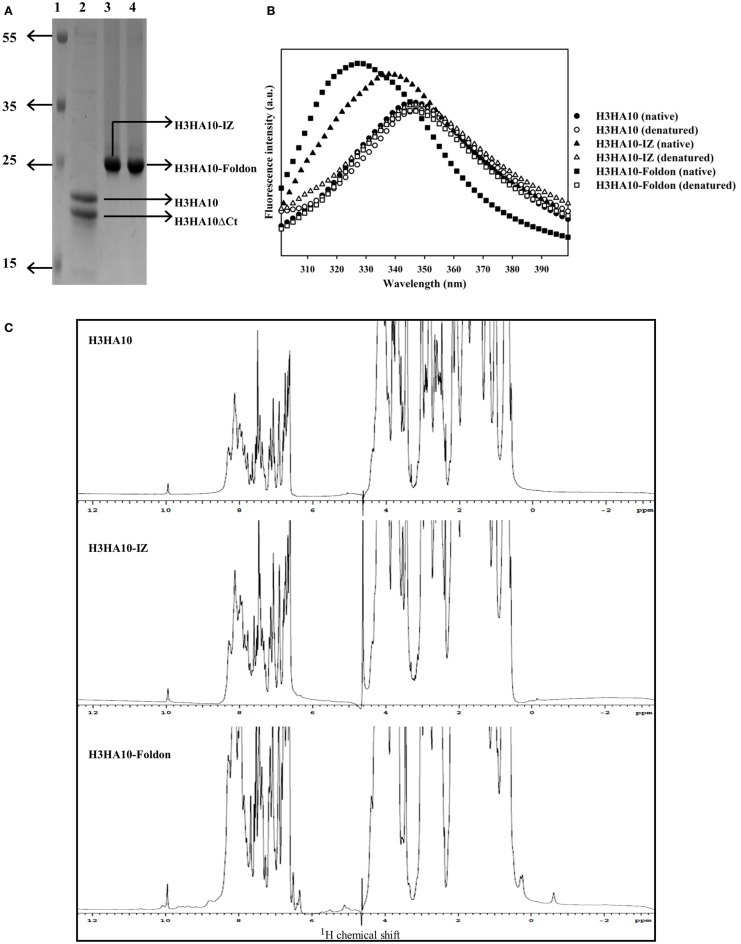
**Protein purification and biophysical characterization of HA stem-fragment immunogens**. **(A)** SDS-PAGE profile of the purified proteins. Lane 1: PageRuler Plus prestained protein ladder (Thermo Scientific), lane 2: H3HA10, lane 3: H3HA10-IZ, and lane 4: H3HA10-Foldon. All the designed proteins were purified from the soluble fraction of *E. coli* cell culture lysate. H3HA10 was partially degraded upon purification. A C-terminal cleavage of 1367.4 Da (determined by mass spectrometry) was observed (H3HA10ΔCt = 16335.9 Da). The derivative(s) of H3HA10 with C-terminal trimerization motif(s) were resistant to *in situ* protein degradation. The migration of the purified proteins in a SDS-PAGE is marginally retarded. The SDS-PAGE gel was stained with Coomassie. **(B)** Fluorescence emission spectra of HA stem-fragment immunogens were recorded under native (PBS, pH 7.4) or denaturing conditions (7M GdmCl in PBS, pH 7.4) as indicated. Unlike H3HA10, both H3HA10-IZ and H3HA10-Foldon showed a significant red-shift in the emission maxima upon denaturation indicating a compact tertiary conformation. **(C)** 1D ^1^H NMR spectra of HA stem-fragment immunogens. The improved chemical shift dispersion in the upfield (0.5–1.0 ppm) and/or downfield (9–11 ppm) regions of the ^1^H NMR spectra of H3HA10-IZ and H3HA10-Foldon is consistent with the fluorescence data, indicating that trimerization motifs assist in the folding of the HA stem in the absence of the transmembrane (TM) domain, with H3HA10-Foldon appearing more structured than H3HA10-IZ.

Recombinant protein expression in prokaryotic systems is economical and amenable for rapid production. However, prokaryotic expression of heterologous viral proteins in native-like conformation has been challenging. Human pathogenic viruses hijack the host protein machinery for synthesis and undergo post-translational modifications (PTMs). Influenza proteins expressed in *E. coli* lack PTMs and can potentially aggregate. Previous efforts at bacterial expression of HA resulted in inclusion bodies and entailed refolding (28, 30, 49). However, in this study, all of our designed immunogens were purified from the soluble fraction of the *E. coli* cell culture lysate. We obtained modest protein yields of ~10-15 mg/l of the culture, using unoptimized shake-flask cultures. We could achieve ≥95% purity as assayed by SDS-PAGE using a single affinity-purification step (Figure 2A). We did not observe any higher order impurities. The purity of the protein was also confirmed using ESI-MS over a mass range of 10–200 kDa.

Surprisingly, partial degradation of H3HA10 was observed upon purification. The addition of protease inhibitor cocktail tablet (cOmplete ULTRA Tablets, Roche) during purification did not prevent this. A C-terminal cleavage of 1367.4 Da (H3HA10ΔCt = 16335.9 Da) was confirmed by mass spectrometry. It has been previously shown that cellular proteases can degrade protein segments with extended conformations *in situ* (50). We hypothesized that C-terminal conjugation of H3HA10 with a synthetic trimerization motif might abate *in situ* protein degradation. Encouragingly, we observed that the addition of either IZ or foldon domains could completely curtail protein degradation *in situ* (Figure 2A).

The integrity of the protein hydrophobic core was probed by intrinsic fluorescence measurements. H3HA10 did not exhibit red-shift in the emission maximum upon denaturation with GdmCl, indicating an extended conformation which may explain the observed *in situ* protein degradation. In contrast, both H3HA10-IZ and H3HA10-Foldon showed significant red-shift in the emission maxima upon denaturation, indicating a compact tertiary conformation (Figure 2B). These results were further substantiated by the one-dimensional ^1^H-NMR spectrum of the designed immunogens. The C-terminal trimerization motifs assist the folding of H3HA10. Both H3HA10-IZ and H3HA10-Foldon have improved solution properties as inferred from resolved resonance lines in the upfield (0.5–1.0 ppm) and/or downfield (9–11 ppm) regions of the ^1^H-NMR spectrum (Figure 2C). The upfield shifted signals result from interactions between methyl protons that are spatially close to buried aromatic rings in the hydrophobic core.

**Figure 3:**
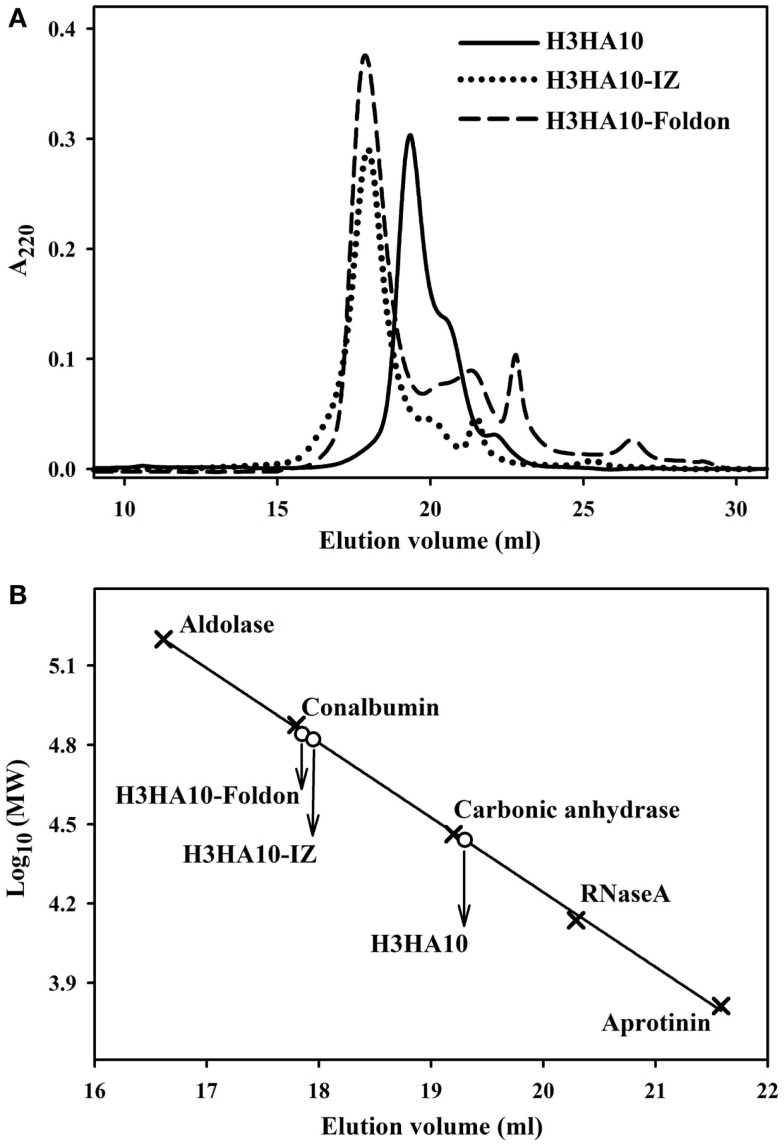
**Oligomeric state of “headless” HA stem immunogens**. **(A)** Size-exclusion chromatography of the purified proteins was done at room temperature under non-denaturing conditions using a buffer (PBS, pH 7.4) equilibrated analytical Superdex-200 column. The disproportionate retention of H3HA10 because of an extended conformation (inferred from fluorescence and 1D ^1^H-NMR measurements) impedes accurate molecular weight estimation. The shoulder of the elution peak corresponds to H3HA10ΔCt. H3HA10-IZ and H3HA10-Foldon predominantly elute as a homogenous oligomer (probably trimer) and do not form higher order aggregates. **(B)** The column was calibrated using a broad range of molecular weight markers (x). The elution volume(s) of the designed protein(s) corresponding to A_220_ signal maxima were plotted [open circles (∘)] on the calibration curve to estimate the molecular weights.

The core of HA stem is assembled by three long, α-helical, parallel coiled-coils. The recapitulation of native HA contacts would promote the trimerization of HA stem mimetics. We determined the oligomeric state of the designed HA stem-fragment proteins using gel-filtration chromatography. The extended conformation of H3HA10 (inferred from fluorescence and ^1^H-NMR measurements) impedes accurate molecular weight estimation from size exclusion chromatography because of disproportionate retention along the column. However, the protein probably elutes as a monomer. The shoulder of the elution peak corresponds to H3HA10ΔCt. In contrast, H3HA10-IZ and H3HA10-Foldon predominantly elute as a homogenous oligomer (probably trimer) and do not form higher order aggregates (Figures 3A,B). This is consistent with previous reports which showed that trimerization motifs facilitate the oligomerization of ΔTM (transmembrane domain deleted) HA stem (32). The molecular weight (~72.5 kDa) of the oligomer estimated from gel-filtration is marginally higher, but close to the theoretical molecular weight of a trimer (∼21.5 × 3 = 64.5 kDa). The discrepancy in the aforementioned molecular weight estimates of the oligomer arises possibly because the designed HA stem-fragment proteins are not globular.

The pan-influenza neutralizing antibody FI6 is selective in binding exclusively the pre-fusion conformation of HA. FI6 binds a conformation sensitive epitope in the HA stem that is disrupted by the structural re-arrangement of HA in the post-fusion conformation (25). Therefore, binding of the designed “headless” stem immunogens to FI6 is an infallible quality control of their conformation. The HA stem-fragment immunogen H3HA10 bound FI6v3-scFv with sub-micromolar affinity (412.4 ± 11.6 nM) (Table 1; Figure S2 in Supplementary Material). On the other hand, full-length rHA (H3 A/Aichi/2/68) bound FI6v3-scFv with very high affinity (22.1 ± 2.3 nM). There are several factors that could contribute to the weaker binding of H3HA10 to FI6v3-scFv. Primarily, the designed stem immunogen includes only a subset (~47%) of the FI6 epitope. Next, the aggregation state of H3HA10 in solution (monomer) could contribute to the slower *k*_on_ and higher *k*_off_ in comparison to the trimeric, full-length rHA (Table 1; Figures S2 in Supplementary Material). Accordingly, the oligomeric derivatives of H3HA10 had considerably tighter binding to FI6v3-scFv. H3HA10-IZ and H3HA10-Foldon had an equilibrium dissociation constant (*K*_D_) of 89.5 ± 3.2 and 114.3 ± 6.8 nM, respectively (Table 1; Figures S2 in Supplementary Material).

**Table 1:** **HA stem-fragment immunogens bind conformation specific bnAb**.

Analyte	FI6v3-scFv^a^		
	*k*_on_ (M^–1^s^–1^)	*k*_off_ (s^–1^)	*K*_D_ (nM)
H3HA10	8.11 × 10^3^	3.41 × 10^–3^	412.4 ± 11.6
H3HA10-IZ	1.01 × 10^4^	9.03 × 10^–4^	89.5 ± 3.2
H3HA10-Foldon	9.27 × 10^3^	1.06 × 10^–3^	114.3 ± 6.8
H3 A/Aichi/2/68 rHA	2.29 × 10^4^	5.07 × 10^–4^	22.1 ± 2.3
H1 A/Puerto Rico/8/34 rHA	1.93 × 10^5^	2.22 × 10^–3^	11.5 ± 1.3

*^a^750 RU of FI6v3-scFv was immobilized on the surface of a CM5-chip*.

*The kinetic parameters for binding were determined by surface plasmon resonance (SPR)*.

### Characterization of antigen-specific antibody response

**Figure 4:**
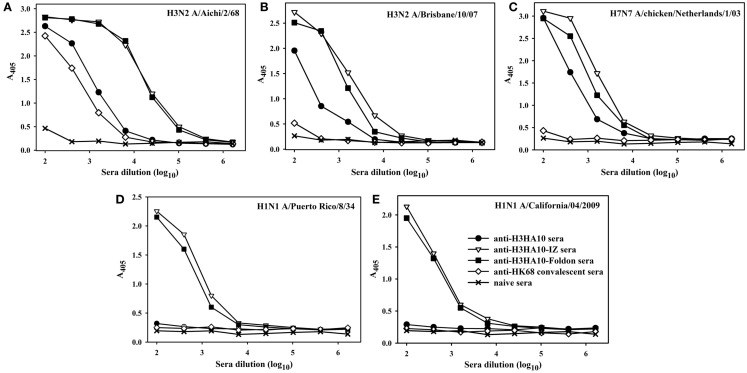
**The designed immunogens elicit broadly cross-reactive, anti-HA antibodies in mice**. The antigen-specific antibody response of the pooled sera (*n* = 10 mice/group, collected 2 weeks after boost) was evaluated in an ELISA with full-length rHA proteins: **(A)** H3N2 A/Aichi/2/68, **(B)** H3N2 A/Brisbane/10/07, **(C)** H7N7 A/chicken/Netherlands/1/03, **(D)** H1N1 A/Puerto Rico/8/34, and **(E)** H1N1 A/California/04/2009. The oligomeric derivatives of H3HA10 induced a higher cross-reactive anti-HA antibody response in comparison to mice that received a sub-lethal, protective dose (0.1LD_90_) of mouse-adapted HK68-virus (convalescent sera).

All the designed HA stem-fragment proteins were highly immunogenic in mice and elicited a robust antibody response with self-titers ≥1 × 10^6^. The antibody titers against the conserved HA stem following a primary infection is lower than the titers elicited against the immunodominant, variable head domain. The predominant antibody response post-infection/vaccination is against the canonical antigenic sites in the globular head domain of HA (1). We assayed antibody binding to full-length rHAs to determine the breadth of antigen (Ag) elicited response. The homologous anti-HA titer was determined using H3N2 A/Aichi/2/68 HA that is nearly identical (99.6%) to H3N2 A/HK/1/68 HA. The anti-HK68 convalescent sera had a homologous anti-HA titer of ~2.5 × 10^4^, but extremely low/undetectable heterologous anti-HA titers suggesting a head-specific response. In contrast, the oligomeric stem-fragment immunogens elicited modest titers of cross-reactive, anti-HA antibodies, validating our design rationale (Figures 4A–C). We achieved a higher cross-reactive anti-HA titer by focusing the antibody response to the HA stem through successful engineering of the conserved HA stem-fragments (Table S1 in Supplementary Material).

The structural divergence in HA stem between influenza groups 1 and 2 establishes distinct group-specific antibody binding profiles. The HA stem directed bnAbs CR6261, F10, and C179 neutralize influenza A group 1 viruses exclusively (4, 24, 44). Binding of these bnAbs to group 2 HAs is abolished because of an N-linked glycan at residue N38_1_ (HA1 subunit), which is highly conserved (6). The glycan shields the conserved eptiope on the HA stem, thereby abrogating the neutralization activity of these bnAbs against influenza A group 2 viruses. A “universal” influenza vaccine must breach this group-restricted antibody response. Extensive screening of over 13,000 monoclonal antibodies (over 90% were influenza-specific) from an individual donor led to isolation of the bnAb FI6 whose epitope overlaps with that of group 1 specific stem-directed bnAbs (25). Binding of the bnAb FI6 to H3 HA is enabled by reorientation of the N-linked glycan at residue N38_1_ upon antibody approach (25). The proteins designed in this study were purified from *E. coli*, and hence lack PTMs like N-linked glycosylation. We therefore hypothesized that non-glycosylated HA stem immunogens mimicking the native, pre-fusion conformation of HA could elicit antibodies which bind HAs belonging to both groups 1 and 2. Encouragingly, sera elicited by H3HA10-IZ and H3HA10-Foldon had detectable antibody titers against divergent H1 HAs belonging to group 1 (Figures 4D,E).

The stem-directed neutralizing antibodies interfere with the establishment of viral infection by inhibiting membrane fusion. These antibodies do not prevent virus attachment to host cell receptors detected in a hemagglutinin inhibition (HI) assay (51). We measured the serum neutralization titers using a pseudotyped virus particle entry inhibition assay (40). The sera elicited by HA stem-fragment immunogens showed significant entry inhibition of the heterologous H3 A/Wisconsin/67/2005 (with A/Udorn/307/1972 N2) influenza pseudotyped virus (Table 2), above the background of the naïve sera (Figure S3 in Supplementary Material). The reason(s) for the high background with the naïve mice sera are not well understood.

**Table 2:** ***In vitro* pseudotyped virus particle entry inhibition with HA stem immunized mice sera**.

Immunogen	IC_50_^a^
H3HA10	13479
H3HA10-IZ	9082
H3HA10-Foldon	16935
Naïve	8287

*^a^IC_50_ titer is the reciprocal of the sera dilution at which half-maximal entry inhibition was observed. Sera were collected 14 days after the boost and pooled*.

*The antibodies elicited by HA stem-fragment immunogens inhibited the entry of a heterologous H3 A/Wisconsin/67/2005 (with A/Udorn/307/1972 N2) influenza pseudotype virus*.

### HA stem-fragment immunogens confer partial protection *In vivo*

**Figure 5:**
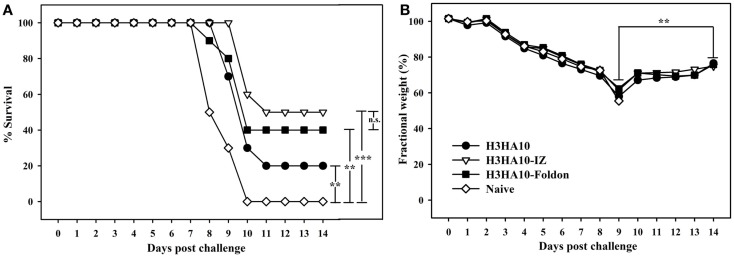
***In vivo* efficacy of HA stem-fragment immunogens against lethal homologous virus challenge**. Mice (*n* = 10/group) were vaccinated with the test immunogens on days 0 (prime) and 28 (boost). The immunized mice were challenged 3 weeks after boost with 2LD_90_ of mouse-adapted HK68 virus. **(A)** Survival and **(B)** average weight changes (of surviving mice) were monitored for 14 days post virus challenge. Naïve mice were used as controls. The oligomeric derivatives of H3HA10 conferred partial protection (40–50%). However, all the surviving mice showed significant weight recovery by end of the study period after initial weight loss. The efficacy of test immunogens was evaluated by analyzing the Kaplan–Meier survival curves with log rank significance test (*p*-values: *** ≤0.005, ** ≤0.05, and n.s. ≥ 0.05). The difference(s) in fractional body weight(s) after recovery (at day 14) and maximal loss (day 9) were analyzed by Student’s *t*-test (*p*-value: ** ≤0.05).

We evaluated the *in vivo* efficacy of HA stem-fragment immunogens against lethal viral infection. Mice were challenged intranasally with a lethal dose (2LD_90_) of homologous mouse-adapted H3N2 A/HK/1/68 virus 3 weeks after the secondary immunization (boost). H3HA10 conferred minimal protection (20%) against virus challenge. Although, the oligomeric derivatives of H3HA10 elicited cross-reactive, anti-HA antibodies, they conferred only partial protection (40–50%) (Figure 5A). However, all surviving mice showed significant weight recovery by the end of the study period after initial weight loss (Figure 5B).

## Discussion

The emergence of “novel” influenza virus strains with the potential to cross-over the species barrier and infect humans has raised alarms across the global health surveillance system. The limitations of current vaccines, namely, strain-specific protection and lengthy production time, have necessitated the development of novel vaccines (1).

A primary influenza infection/vaccination results in an antibody response biased toward the immunodominant, hyper-variable antigenic sites in the globular head domain of HA (43). Alternative vaccination strategies have been adopted to skew the humoral response in favor of the conserved HA stem which can potentially increase the breadth of protection. A vaccination regimen with repeated DNA and/or protein immunizations with full-length, chimeric HAs was shown to enhance the stem-directed antibody response (52). Full-length HA displayed on ferritin-nanoparticles elicited stem-directed antibodies in addition to a robust head-directed response (53). HA N-linked glycosylation has also been engineered to induce cross-strain protection against influenza infection (54, 55).

In alternate approaches, the HA stem has been stabilized in the absence of the globular head domain. We have previously demonstrated that site-specific charged (Asp) mutations can destabilize the post-fusion conformation of the HA stem and shift the equilibrium toward the metastable, pre-fusion conformation at neutral-pH (28). The bacterially expressed HA stem conferred complete protection against virus challenge in mice. However, these proteins formed inclusion body aggregates upon expression and required refolding. The refolded proteins were aggregation prone. In subsequent studies, we engineered HA stem sub-structures to elicit a stem-specific immune response (32, 56). We successfully designed “headless” HA stem-fragment immunogens from influenza A group 1 viruses which were purified from the soluble fraction in *E. coli*. These thermotolerant, trimeric immunogens conferred robust subtype-specific protection *in vivo* (32).

Structural divergence in the stem-domain of HA between groups 1 and 2 results in the group-specific neutralization profile of various stem-directed bnAbs (6). In lieu of a “universal” influenza vaccine, a composite of immunogen(s) from both groups is a practical alternative. Herein, we report the characterization of HA stem-fragment immunogens designed from the H3N2 strain (A/Hong Kong/1/68) that caused the “1968 influenza pandemic”.

We intended to enhance the breadth of Ag-specific immune response by targeting the conserved regions of HA. An exhaustive analysis of full-length H3 HA sequences revealed multiple sub-structures within the HA stem that are conserved. These conserved sub-structures form discrete epitopes that are targeted by different bnAbs: the pan-influenza bnAb FI6 binds the epitope lined by residues of the A-helix (HA2-subunit) (25), while the viral membrane proximal β-sheet lines the epitope of group 2 specific bnAbs CR8020 and CR8043 (5, 27). Although, cryo-EM studies demonstrated that HA trimers on the virion surface could complex with a stem-directed bnAb (44), the relative accessibility of HA stem sub-structures on the crowded virion surface may influence the *in vivo* efficacy of vaccine candidates targeting these epitopes separately. The designed HA stem-fragment immunogen, H3HA10, comprises a subset (~47%) of the bnAb FI6 epitope and completely lacks the epitopes for CR8020 and CR8043. The interaction network of the HA stem was minimally perturbed to mimic the native, pre-fusion conformation in the designed immunogen. Further, the C-terminal conjugation of H3HA10 with a trimerization motif (IZ/foldon) improved the solution properties of the protein. Soluble prokaryotic (*E. coli*) expression of the designed immunogens enables rapid production. Although the oligomeric derivatives of H3HA10 elicited cross-reactive anti-HA antibodies that inhibited entry of a heterologous H3 HA pseudotyped virus *in vitro*, they conferred only partial protection (40–50%) after virus challenge in mice. There is disconnect between the high entry inhibition IC_50_ values (Table 2) and the lack of a robust protective response (Figure 5A) elicited by the designed immunogens. The high entry inhibition IC_50_ values are likely to be the consequence of using a highly sensitive entry inhibition assay. The lack of a strong correlation between the entry inhibition IC_50_ values and survival warrants further investigation into the role of antibody-dependent effector functions such as antibody-dependent cell-mediated cytotoxicity (ADCC) and other Ag-specific antiviral mechanism(s). There is considerable scope to improve our current design to enhance the *in vivo* efficacy, for instance, by incorporating a larger footprint of the bnAb FI6 epitope.

Although the H3HA10 series of immunogens described here are expressed in soluble form and are not aggregation prone, they had relatively lower *in vivo* efficacy as compared to our previously designed HA stem immunogen (H3HA6). This is probably because H3HA6 includes the entire HA stem presenting distinct epitopes of multiple bnAbs to the immune system (28). The oligomeric derivatives of the designed HA stem immunogens elicited a robust antibody response against the homologous H3N2 HA. While these antibodies were cross-reactive, the titers against heterologous H3 and H7 HAs were 10-fold lower. The elicited antibodies also exhibited weak cross-group (group 1 HAs) reactivity. The antibody-HA reactivity profile correlates well with the residue conservation between the influenza strains evaluated in our assay (Table S1 in Supplementary Material). Therefore, we hypothesize that increasing the footprint of bnAb epitopes in the designed immunogen can further improve the binding profile. A comparison with the designed HA stem-fragment immunogen, H1HA10 (32), from influenza A group 1 viruses reaffirms the necessity to include a larger footprint of bnAb epitopes. H1HA10 includes ~70% of the pan-influenza neutralizing bnAb F16 epitope, while H3HA10 includes only ~47% of the epitope. Further design optimization will explore mutations to increase the strength of inter-protomer interactions. For example, a recent study demonstrated that engineered Cys mutations in the LAH of the HA2-subunit of pandemic HA (H1N1 A/California/2009) promote the formation of covalent trimers (31). Despite these lacunae, the immunogens described in this study do provide partial protection against lethal pathogenic challenge and elicit broadly cross-reactive HA stem-directed antibodies. Our studies provide a framework for the design of future influenza A group 2 HA stem-fragment immunogens.

## Author Contributions

VM, XL, and RV designed the experiments. VM, MC, FF, and NT performed the experiments. All authors analyzed the data and assisted in manuscript preparation. VM, JF, and RV wrote the manuscript.

## Conflict of Interest Statement

The authors declare that the research was conducted in the absence of any commercial or financial relationships that could be construed as a potential conflict of interest.

## Supplementary Material

The Supplementary Material for this article can be found online at http://journal.frontiersin.org/article/10.3389/fimmu.2015.00329

## Footnotes

^1^http://www.cdc.gov/

^2^http://www.ncbi.nlm.nih.gov/genomes/FLU/Database/nph-select.cgi?go=database
