# Supplementary material for: Hemagglutinin Sequence Conservation Guided Stem Immunogen Design from Influenza A H3 Subtype
Source: Front Immunol. 2015 Jun 26;6:329. doi: 10.3389/fimmu.2015.00329 (PMC4481277; doi:10.3389/fimmu.2015.00329)
Supplement: Supplementary file 1 [file Table_1.PDF]

Table S1: HA residue conservation across representative strains of influenza A subtypes (H1-H16). The conservation score across all the analyzed HA sequences is greater within the designed HA stem-fragment immunogen than in full-length HA.

**Strain**	**% identity with full-length H3N2 A/Hong Kong/1/68 HA**	**% identity with the designed stem-fragment H3HA10**
Group 1 HAs		
A/duck/Vietnam/OIE-2334/2010 (H9)	39.3	42.3
A/mallard/Minnesota/Sg-00570/2008 (H8)	40.1	46.2
A/ruddy turnstone/Delaware/Sg-00540/2008 (H12)	42.2	47.0
A/duck/Thailand/CU5388/2009 (H11)	40.5	47.7
A/gull/Maryland/704/1977 (H13)	38.2	43.1
A/mallard/Quebec/02916-1/2009 (H16)	37.4	45.4
A/duck/Guangxi/038/2009 (H6)	42.1	47.0
A/Puerto Rico/8/34 (H1)	40.9	43.1
A/California/04/2009 (H1)	42.6	47.7
A/Albany/20/1957 (H2)	41.1	46.2
A/Viet Nam/1203/2004 (H5)	41.5	43.9
Group 2 HAs		
A/wild duck/Korea/7-D19/2005 (H4)	65.8	80.8
A/mallard/Astrakhan/263/1982 (H14)	65.4	80.0
A/Aichi/2/1968 (H3)	99.7	100.0
A/Brisbane/10/2007 (H3)	87.3	95.4
A/American black duck/New Brunswick/00471/2010 (H10)	49.7	60.0
A/chicken/Netherlands/1/03 (H7)	46.8	60.0
A/duck/Australia/341/1983 (H15)	47.2	57.7
