# Supplementary material for: Hemagglutinin Sequence Conservation Guided Stem Immunogen Design from Influenza A H3 Subtype
Source: Front Immunol. 2015 Jun 26;6:329. doi: 10.3389/fimmu.2015.00329 (PMC4481277; doi:10.3389/fimmu.2015.00329)
Supplement: Supplementary file 2 [file Image_1.PDF]

Supplementary information

(1) H3HA10

A**S**PNGTLVKTITDDQIEVTNATELVQSS**GSAGSA**NDKPFQN**T**NK**R**T**S**GASPKYVKQNTLKLA
TG**Q**R**GSAGSA**A**T**DQINGKLNRVIEKTNEK***D***HQIEKEFSE***D***EGRIQDLEKYVEDTKIDLWSYN
AELLVALENQHTIDLTDS

(2) H3HA10-IZ

A**S**PNGTLVKTITDDQIEVTNATELVQSS**GSAGSA**NDKPFQN**T**NK**R**T**S**GASPKYVKQNTLKLA
TG**Q**R**GSAGSA**A**T**DQINGKLNRVIEKTNEK***D***HQIEKEFSE***D***EGRIQDLEKYVEDTKIDLWSYN
AELLVALENQHTIDLTDSQGT**GG***IKKEIEAIKKEQEAIKKKIEAIEKEIEA*

(3) H3HA10-Foldon

A**S**PNGTLVKTITDDQIEVTNATELVQSS**GSAGSA**NDKPFQN**T**NK**R**T**S**GASPKYVKQNTLKLA
TG**Q**R**GSAGSA**A**T**DQINGKLNRVIEKTNEK***D***HQIEKEFSE***D***EGRIQDLEKYVEDTKIDLWSYN
AELLVALENQHTIDLTDSQGT**GG***GYIPEAPRDGQAYVRKDGEWVLLSTFL*

Figure S1: Sequences of the designed HA stem-fragment immunogens. H3HA10 was designed from the H3N2 A/Hong Kong/1/68 HA sequence (GenBank Accession: AAK51718.1) deposited at NCBI-Flu Database. H3HA10 included conserved HA stem-fragments 19_1-46_1 (HA1), 290_1-321_1 (HA1) and 44_2-113_2 (HA2). Mutations were incorporated to re-surface hydrophobic patches exposed during protein minimization (bold and underlined). Mutations ($F63_2D$ and $V73_2D$) were introduced to destabilize the low-pH conformation of HA (bold and italics). $Cys305_1$ was mutated to Ser to prevent inter-molecular disulfide bonds. The HA fragments were connected by linkers (bold). Derivatives of H3HA10 with C-terminal trimerization motif(s) (italics) IZ (H3HA10-IZ) and ‘foldon’ (H3HA10-Foldon) were made.
