# Supplementary material for: Hemagglutinin Sequence Conservation Guided Stem Immunogen Design from Influenza A H3 Subtype
Source: Front Immunol. 2015 Jun 26;6:329. doi: 10.3389/fimmu.2015.00329 (PMC4481277; doi:10.3389/fimmu.2015.00329)
Supplement: Supplementary file 3 [file Image_2.PDF]

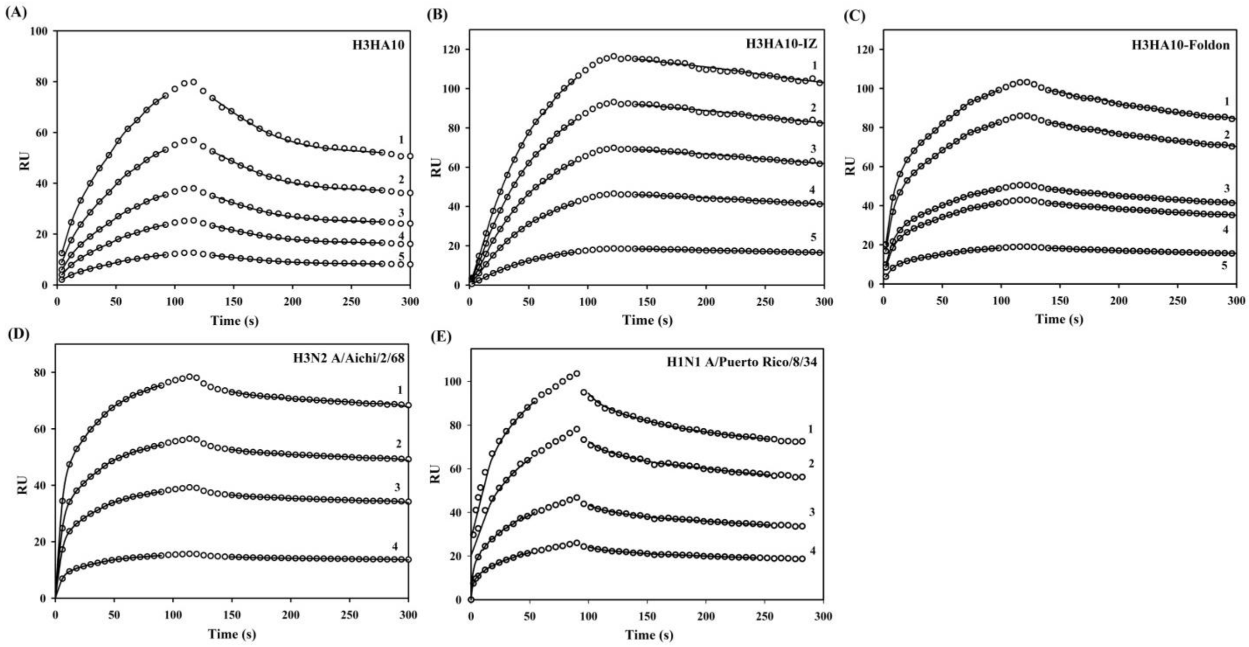

Figure S2: Binding sensograms of HA stem-fragment immunogens and full-length rHAs to FI6v3-scFv. A CM5-chip sensor channel was immobilized with 750 RU of FI6v3-scFv. A concentration series of the analytes were passed over the immobilized ligand to determine the kinetic parameters (Table 1). **(A)** H3HA10 (Trace 1-5: 7500nM, 5000nM, 2500nM, 1000nM and 500nM). **(B-C)** H3HA10-IZ and H3HA10-Foldon (Trace 1-5: 2500nM, 1000nM, 750nM, 500nM and 250nM). **(D-E)** H3N2 A/Aichi/2/68 and H1N1 A/PR/8/34 rHAs (Trace 1-4: 150nM, 75nM, 25nM and 10nM). The kinetic parameters were obtained by a global fitting of the data to the 1:1 Langmuir interaction model using BIA EVALUATION 3.1 software. The data points are in open circles, while the fits are represented by solid lines.
