# Supplementary material for: Hemagglutinin Sequence Conservation Guided Stem Immunogen Design from Influenza A H3 Subtype
Source: Front Immunol. 2015 Jun 26;6:329. doi: 10.3389/fimmu.2015.00329 (PMC4481277; doi:10.3389/fimmu.2015.00329)
Supplement: Supplementary file 4 [file Image_3.PDF]

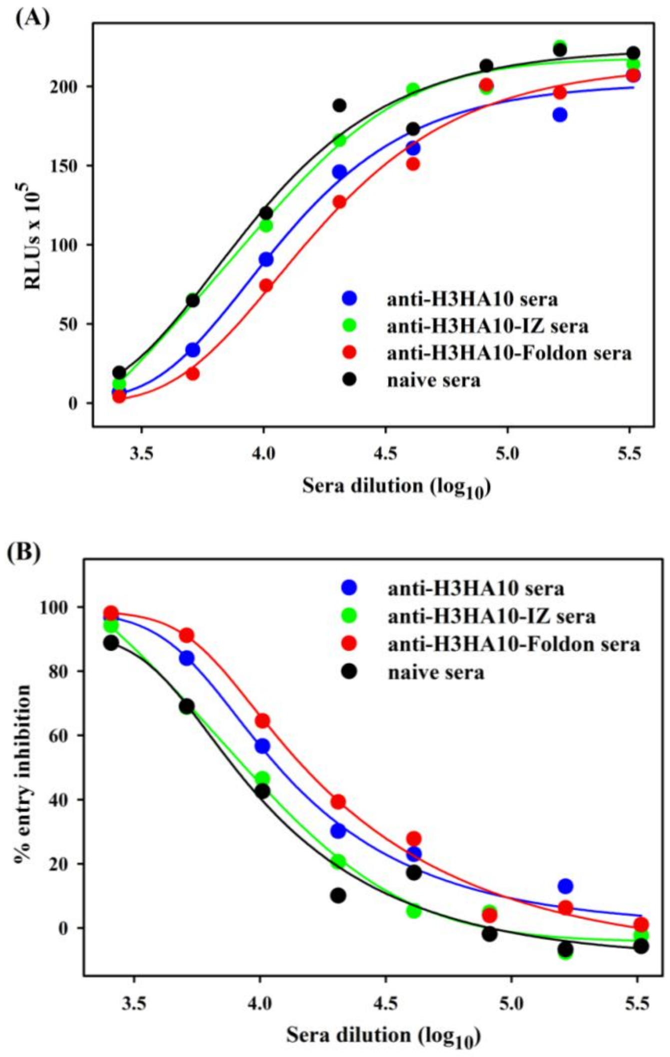

Figure S3: H3 A/Wisconsin/67/2005 (with A/Udorn/307/1972 N2) pseudotyped virus particle entry inhibition with HA stem immunized mice sera. Serial dilutions of heat-inactivated sera were incubated with 2×10^7 relative luminescence units (RLUs) of pseudotypes/well at 37°C for 1h. After incubation, 1.5×10^4 HEK293T cells were added to each well and incubated for another 48h at 37°C. The pseudotyped virus particle entry was monitored by luminescence signals. Inhibition curves **(A)** before normalization and **(B)** after normalization are shown here. The data points are in circles, while the fits are represented by solid lines.
